# Can species‐specific prey responses to chemical cues explain prey susceptibility to predation?

**DOI:** 10.1002/ece3.4000

**Published:** 2018-04-10

**Authors:** Marek Šmejkal, Daniel Ricard, Zuzana Sajdlová, Martin Čech, Lukáš Vejřík, Petr Blabolil, Ivana Vejříková, Marie Prchalová, Mojmír Vašek, Allan T. Souza, Christer Brönmark, Jiří Peterka

**Affiliations:** ^1^ Biology Centre of the Academy of Sciences of the Czech Republic Institute of Hydrobiology České Budějovice Czech Republic; ^2^ Faculty of Science University of South Bohemia České Budějovice Czech Republic; ^3^ Department of Aquatic Ecology Lund University Lund Sweden; ^4^ Fisheries and Oceans Canada Gulf Fisheries Centre Moncton Canada

**Keywords:** chemical communication, predator‐prey interaction, schreckstoff, wels

## Abstract

The perception of danger represents an essential ability of prey for gaining an informational advantage over their natural enemies. Especially in complex environments or at night, animals strongly rely on chemoreception to avoid predators. The ability to recognize danger by chemical cues and subsequent adaptive responses to predation threats should generally increase prey survival. Recent findings suggest that European catfish (*Silurus glanis*) introduction induce changes in fish community and we tested whether the direction of change can be attributed to differences in chemical cue perception. We tested behavioral response to chemical cues using three species of freshwater fish common in European water: rudd (*Scardinius erythrophthalmus*), roach (*Rutilus rutilus*), and perch (*Perca fluviatilis*). Further, we conducted a prey selectivity experiment to evaluate the prey preferences of the European catfish. Roach exhibited the strongest reaction to chemical cues, rudd decreased use of refuge and perch did not alter any behavior in the experiment. These findings suggest that chemical cue perception might be behind community data change and we encourage collecting more community data of tested prey species before and after European catfish introduction to test the hypothesis. We conclude that used prey species can be used as a model species to verify whether chemical cue perception enhances prey survival.

## 1 INTRODUCTION

Predator‐prey interactions are key forces structuring communities in both terrestrial and aquatic environments (Crooks & Soulé, 1999; Mills, Soulé, & Doak, 1993; Sinclair, Mduma, & Brashares, 2003). Prey may respond to predation threats by migrating into low‐predation habitats (Chapman et al., 2012) or by employing an adaptive behavioral response that prevents the predator from successfully finishing the predation cycle (Apfelbach, Blanchard, Blanchard, Hayes, & McGregor, 2005; Lima & Bednekoff, 1999). A population of prey that does not adjust its habitat or behavior to its predators may strongly decline as a consequence (Hölker et al., 2007; Short, Kinnear, & Robley, 2002).

To decrease the risk of mortality, prey should minimize the probability of encounter with their predators, their appeal to predators and, further, prey should maximize their escape probability (Johansson, Turesson, & Persson, 2004; Turesson & Persson, 2002). Early predator detection lowers the probability of direct encounter and increases the probability of escape during an attack due to enhanced vigilance (Brown, Poirier, & Adrian, 2004). Therefore, this ability is regarded as essential to increased survival, especially for prey relying on escape abilities rather than morphological defenses (Krause & Godin, 1992; Lima & Bednekoff, 1999; Miller & Surlykke, 2001).

In aquatic systems, chemical cues are recognized as being highly important for predator detection and in determining prey survival (Mirza & Chivers, 2000). Among many fish genera, warning signals are mediated by chemical cues released into the water by skin damage (alarm cue) or via digestive cues excreted by predators, which contain cues of digested conspecifics (Ferrari, Wisenden, & Chivers, 2010). Both chemical alarm cues and diet cues can be used to recognize a novel predator as a risk when they are encountered at the same time as the predator odor (Brown & Smith, 1998; Chivers & Smith, 1994; Ferrari, Messier, & Chivers, 2008). Predator odor is then identified as a threat even though it is no longer accompanied by chemical alarm/diet cues, and prey retain the anti‐predator response for a long period of time (Ferrari, Messier, & Chivers, 2006; Pettersson, Nilsson, Bronmark, & Brönmark, 2000). This system of learning provides the ability to associate novel species as predators as well as to avoid environments with a high concentration of alarm, diet or predator odor cues. The prey response to alarm and dietary cues can be represented by short‐term behavioral changes decreasing the probability of encounter with predators (e.g., increase in shoaling cohesiveness, use of refuge use or decrease in fish activity; Chivers & Smith, 1994; Pollock, Chivers, Mirza, & Wisenden, 2003). Furthermore, long‐term changes in body morphology may facilitate higher survival chances: crucian carp (*Carassius carassius*) increase its body depth in environment containing chemical cues, and its deeper body provides higher escape probability after encounter with predator (Brönmark & Miner, 1992; Brönmark & Pettersson, 1994; Domenici, Turesson, Brodersen, Brönmark, & Bronmark, 2008). Regardless of the species‐specific reaction, a well‐developed perception of danger and well‐chosen anti‐predator strategies should lead to increased survival at the population level.

The aim of this study was to test the ability to detect and react to dangers represented by European catfish chemical cues (*Silurus glanis*) among three model prey species: perch (*Perca fluviatilis*), roach (*Rutilus rutilus*), and rudd (*Scardinius erythrophthalmus*). Subsequent behavioral changes (activity, shoal cohesion, and use of refuge) were examined in the experiment. Furthermore, we evaluated the prey preferences of catfish when exposed to the same three prey species in an experimental laboratory setting. Finally, we review large‐scale experiment data suggesting that European catfish might trigger change in prey fish community and encourage collecting more field evidence to verify the hypothesis that the direction of change is in accordance with behavioral response to chemical cues of tested prey species.

## 2 MATERIALS AND METHODS

### 2.1 Species studied

Perch, roach, and rudd are widespread in the European part of the Palearctic region and are common prey species of piscivores. With a similar approximate maximal length of 50 cm and a maximum lifespan of under 10 years, these fish species possess similar life histories to some extent and co‐occur in many European lake communities (Hölker et al., 2007; Mehner, Diekmann, Bramick, & Lemcke, 2005). During adulthood, the food niche overlap among these species is not extensive because each species utilizes a different food source. Perch switch to piscivory at a length of approximately 15 cm, while rudd specialize into herbivores and roach utilize various food sources, including detritus (Horppila & Nurminen, 2009; Persson et al., 2003). All prey fish species have an affinity for littoral habitats in the lake; however, roach in particular and rudd also utilize pelagic areas (Eklöv & Hamrin, 1989; Prchalová et al., 2008). The European catfish is a predominantly nocturnal and twilight‐active large predator species (Boujard, 1995; Gjelland et al., 2017). It has recently spread into many non‐native waters and triggered noticeable changes in fish and waterfowl communities (Benejam, Carol, Benito, & García‐Berthou, 2007; Copp et al., 2009; Vejřík et al., 2017). Because of its nocturnal activity and the high twilight activity of prey species, suggesting a high encounter rate between predator and prey (Boujard, 1995; Westin & Aneer, 1987), the experimental protocol was performed under low light conditions. As many fish species detect near‐infrared and infrared light (Matsumoto & Kawamura, 2005; Meuthen, Rick, Thünken, & Baldauf, 2012), we did not use infrared sensitive camera for night observations.

### 2.2 Chemical cue experiments

#### 2.2.1 Test fish

Experimental fish were obtained in the fall of 2014 from a pond inhabited by catfish and pike (*Esox lucius*) near České Budějovice, Czech Republic (48°99′04″ N, 14°43′70″ E), that is, these fish were non‐naïve prey with a natural reaction to catfish odor and diet cues. Altogether, 120 perch (measured in standard length SL = 114 mm ± 6 mm *SD*), 120 roach (109 ± 13), 120 rudd (106 ± 12), and two catfish (750 and 770 mm) were used in the chemical cue experiments I (60 fish of each species) and II (60 fish of each species). Furthermore, 10–15 individuals of each prey species of the same size were obtained to feed the catfish during the study. All fish were acclimatized in the laboratory 2 weeks prior to conducting the experiments. The prey fish were fed ad libitum with a combination of frozen copepods (*Cyclops* sp.) and aquaculture pellets during the acclimation period and the experiments. The light regime was set to 2:10:2:10 hrs (twilight/light/twilight/dark) with the light intensity set at 2/50/2/0 lx. The water temperature was maintained at 18°C.

#### 2.2.2 Stimulus and control preparation

The terminology chemical cues are used in accordance with Pettersson, Andersson, & Nilsson (2001) and Pettersson et al. (2000). Stimulus preparation follows a general methodology adjusted to large catfish size (Brown, Chivers, & Smith, 1996; Mathis & Smith, 1993b, Pettersson et al., 2000). The stimulus preparation simulates large predator feeding regime once in a 24–48 hr (Boujard, 1995; Carol, Zamora, & García‐Berthou, 2007) and attempts to avoid unnaturally strong reaction caused by fresh dietary and alarm cues produced by well‐fed fish, which are less likely to forage (Vejřík et al., 2017).

During the acclimation period, the catfish were held in a 2,500 L pool and fed all three prey fish species. Before the collection of stimuli began, the catfish were deprived of food for 3 days. Afterward, the catfish were fed once a day for 5 days with the prey species to be investigated (roach, rudd, or perch; approximate total prey weight 150 g). Twenty‐four hours after the last feeding, each catfish was rinsed with dechlorinated tap water and placed into a stimulus collection tank (130 × 60 × 65 cm, filled with 230 L of dechlorinated tap water). After another 24 hr, tank water containing chemical cues from both catfish was collected, mixed together to avoid individual catfish effects and frozen at −20°C in 100 ml plastic containers. As a water stimulus control, dechlorinated tap water was frozen in 100 ml plastic containers. The treatment and control water was thawed 1 hr before the experiments.

#### 2.2.3 Testing protocol

Four tanks (60 × 60 cm) filled with 72 L of water were used for the experiments. Each tank had a 15 cm grid drawn on the bottom. Experiments without (I) and with refuge (II) were conducted to evaluate prey fish shoaling, activity, and refuge use. Shoaling and activity were evaluated only in experiment I because the refuge present in experiment II may have influenced fish position and activity. For the refuge treatment, we placed a patch of artificial macrophytes (a plastic plate [20 × 15 cm] with 24 buoyant plastic strips [20 × 2 cm]) in the tank corner. All tanks had a tube placed in the corner (left of the refuge corner) to introduce stimulus/control water as well as an airstone. We ensured that the added stimulus was evenly distributed within one minute by testing the water with the addition of a blue dye. The tanks were monitored from above with a low light‐sensitive camera (SplashCam Delta Vision HD B/W, Ocean Systems).

To decrease stress that individual fish experience when kept alone in a tank we chose to perform the experiments using shoals of three fish per tank. Three randomly chosen individuals of the same species were placed in each of the four tanks 24 hr before the start of the experiment. The experimental fish were used only for a single experiment. Each group of fish was first tested with the control treatment (100 ml of dechlorinated tap water) and then with the predator treatment (100 ml of stimulus‐laden water) in the twilight period of the 24 hr cycle, corresponding to natural dusk. Video recording started 2 min after the 100 ml of stimulus or control water was introduced (to ensure that the added water was evenly distributed) and lasted for 10 min. The predator treatment started 2 min after the control treatment. Twenty control and 20 stimulus trials were conducted per species and experimental setup. The video recordings were evaluated as a blinded experiment. For experiment I, we evaluated shoaling and activity. For shoaling, we used an index that ranged from 1 to 3 as follows: 1 = no fish within one body length of each other, 2 = two fish within one body length of each other, 3 = three fish within one body length of each other. Observations for the shoaling index were recorded every 20 s, for a total of 31 snapshots per replicate. Fish activity was evaluated from the video recording as the number of grid crosses per replicate (a cross was defined as occurring when at least ¾ of a fish's body crossed a grid line). The use of refuge in experiment II was evaluated such that fish presence in the refuge was defined as hiding behavior (four of 16 squares). Refuge use was evaluated as the number of individuals in the refuge every 20 s. The average value of each behavioral variable was determined for each replicate.

### 2.3 Prey selectivity experiment

Non‐naïve experimental fish were obtained from the same pond as above in the spring of 2015. Altogether, 99 perch (SL = 122 mm ± 18 mm *SD*), 99 roach (119 ± 16), 99 rudd (121 ± 18), and seven catfish (714 ± 85) were collected and used in the prey selectivity experiments. All fish were acclimatized in the laboratory for 2 weeks prior to the onset of the experiments. The prey fish were fed ad libitum with a combination of frozen copepods (*Cyclops* sp.) and aquaculture pellets.

The catfish were kept separately during the experimental trials in 4 × 1.5 m tanks filled with 4,200 L water at 19°C. A partial refuge was present in the tank and consisted of a 1 × 1 m plastic plate with 110 buoyant plastic strips (20 × 2 cm). Three fish of each species were placed together in the tank in the evening and left overnight. The prey were size‐matched (length) for each replicate. In the morning, the remaining prey were counted and removed. Each individual prey fish was used only once. Seven replicates were obtained for four catfish individuals, while the remaining three catfish had to be kept together when the experiment was not running due to space limitation in the laboratory. These individuals had to be returned to the pond after 1–2 replicates due to injuries caused by their mutual aggressive behavior. Thirty‐three replicates were obtained in total.

The experimental protocols used in this study were performed in accordance with the guidelines and permission from the Experimental Animal Welfare Commission under the Ministry of Agriculture of the Czech Republic (Ref. No. CZ 01679). All methods were approved by the Experimental Animal Welfare Commission.

### 2.4 Statistical evaluation

#### 2.4.1 Chemical cue experiments

One‐tailed paired t tests or nonparametric Wilcoxon signed‐rank tests were used for analysing changes in behavior when comparing the control and experimental period, depending on normality and variance of the data. A Bonferroni correction was applied for multiple comparisons.

The nonparametric Kruskal–Wallis test was used to compare the behavioral parameters of species in the control treatment to determine possible differences in their basal behavior.

#### 2.4.2 Prey selectivity experiment

The Manly–Chesson selectivity index was computed to evaluate prey vulnerability to predation using the formula:

$$\alpha_{i}=\frac{r_{i}/p_{i}}{\sum_{i=1}^{m}\left(r_{j}/p_{j}\right)},i=1,2,\ldots ,j$$

where *r*_*i*_ is the proportion of food item *i* in the diet, *p*_*i*_ is the proportion of food item *i* in the experiment and *j* is the number of prey species in the experiment (Chesson, 1978). The values of α_*i*_ can range from 0 (complete avoidance) to 1 (complete preference), and with three prey species, α_*i*_ = 0.33 denotes no preference. For the comparison of catfish selectivity, we used repeated measures ANOVA with catfish individuals included as a factor. Because the sphericity of the dependent variables (perch, rudd, and roach selectivity indices) was not satisfied, the differences between the dependent variables were compared by the multidimensional Wilks test. Fisher's LSD test was used for post hoc comparisons.

Statistical analyses were performed in Statistica software (Statistica, Inc., StatSoft, Tulsa, Oklahoma, USA) and R software version 3.2.3 (R Core Team 2015).

## 3 RESULTS

### 3.1 Chemical cue experiments

**Figure 1:**
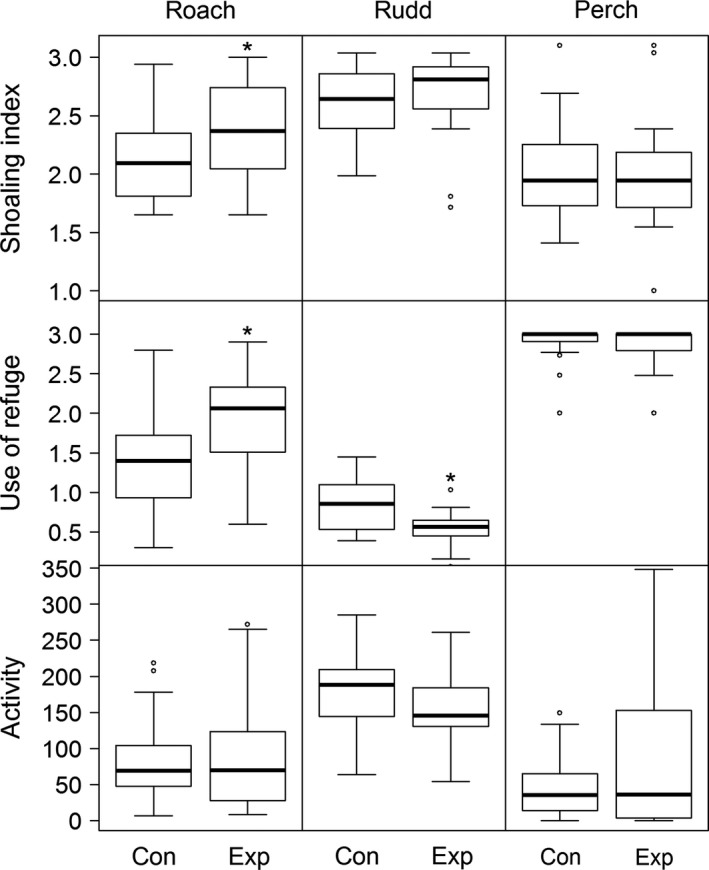
Species‐specific shoaling index (number of fish within one body length of each other), use of refuge (number of fish in the refuge) and activity (number of grid crosses) in control (Con) and chemical cue treatments (Exp), *N* = 20. The boxes represent the boundaries of the upper and lower quartiles, the thick lines represent medians, the whiskers represent 95% confidence intervals and dots are outlying observations. An asterisk indicates a significant difference between the control and the experimental treatment

Each species tested had a different response to the presence of chemical cues. Roach and rudd altered their behavior in the presence of chemical cues compared to the control treatment. Specifically, roach increased their shoaling index (mean ± SD in control vs. experiment, statistics: 2.1 ± 0.3 vs. 2.4 ± 0.4, *t*_(19)_ = −3.09, *p* = .009) and refuge use (1.4 ± 0.7 vs. 1.9 ± 0.6, *t*_(19)_ = −2.55, *p* = .03), while their activity (log_10_) remained unchanged (4.1 ± 0.9 vs. 4.0 ± 1, *t*_(19)_ = 0.32, *p* = .624). In rudd, there was no significant change in the shoaling index (2.5 ± 0.3 vs. 2.6 ± 0.4, *t*_(19)_ = −1.27, *p* = .110) or activity (175 ± 55 vs. 157 ± 53, *t*_(19)_ = 1.27, *p* = .109) in the presence of chemical cues. However, rudd significantly decreased their use of refuges (0.86 ± 0.35 vs. 0.54 ± 0.23, *t*_(19)_ = 2.86, *p* = .015). Perch did not alter any behaviors in the experiment (Wilcoxon signed‐rank test: shoaling index 1.98 ± 0.41 vs. 1.99 ± 0.51, *Z* = 117, *p* = .678; refuge 2.9 ± 0.25 vs. 2.9 ± 0.26, *Z *= 32.5, *p* = .713; activity 47 ± 43 vs. 85 ± 100, *Z* = 80, *p* = 0.826, Figure 1).

All behavioral parameters were significantly different among species in the control treatments: shoaling index (Kruskal–Wallis (K–W) χ^2^ = 19.438, *df* = 2, *p* < .001; rudd 2.52 ± 0.29 > roach 2.10 ± 0.32 > perch 1.98 ± 0.41), refuge use (K–W χ^2^ = 41.89, *df* = 2, *p* < .001; perch 2.89 ± 0.25 > roach 1.39 ± 0.66 > rudd 0.86 ± 0.35), and activity (K–W χ^2^ = 29.68, *df* = 2, *p* < .001, rudd 175 ± 55 > roach 86 ± 63 > perch 47 ± 43; Figure 1).

### 3.2 Prey selectivity experiment

**Figure 2:**
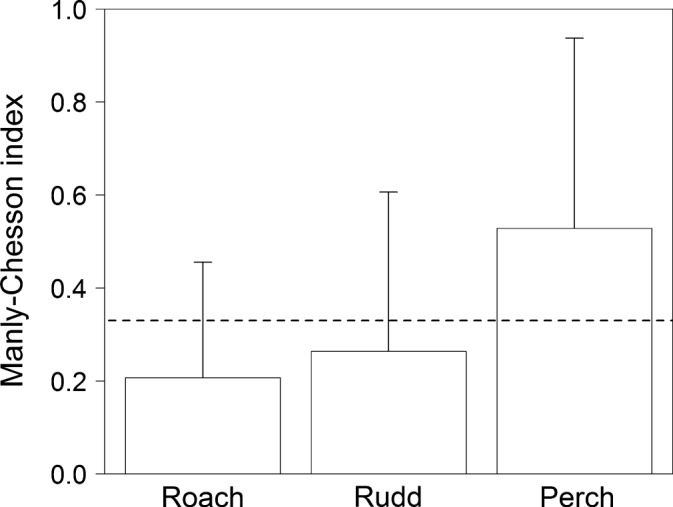
Species‐specific comparison of the Manly–Chesson selectivity index, α (*N* = 33; 7 catfish individuals). Bars represent means and whiskers represent the standard deviations of the multidimensional Wilks model. Dashed line denotes no preference (α = 0.33) for the three species in the experiment

In the prey selectivity experiment, 2.4 ± 1.2 prey fishes were consumed per trial on average. The Manly–Chesson selectivity index was significantly dependent on the prey species (multivariate repeated measures ANOVA, Wilks’ lambda = 0.56, *F*(2,25) = 9.85, *p* < .001). However, post hoc comparisons using Fisher's LSD test did not show any significant differences among species, although perch was preferred over roach at a nearly statistically significant level (perch α = 0.53, rudd α = 0.26, roach α = 0.21; species comparison: rudd vs. roach *p* = .370, rudd vs. perch *p* = .300, roach vs. perch *p* = .055; Figure 2).

## 4 DISCUSSION

The experimental evaluation of the species‐specific reactions to the threat of catfish predation revealed that both roach and rudd were able to detect chemical cues and change their behavior under the threat of predation. However, rudd unexpectedly chose to be more exposed to predation in the experimental treatment compared to the control treatment. We detected no behavioral reaction in perch. We hypothesize that species‐specific catfish detection and avoidance abilities may explain recent findings that roach populations thrive and rudd and perch populations decline in catfish‐stocked lakes (Vejřík et al., 2017).

Roach and rudd markedly differed in their use of refuges in the experimental treatment: while roach increased hiding behavior under the threat of predation, rudd exhibited the opposite reaction. The extent of species‐specific reactions to chemical cues is dependent on their anti‐predator strategy. Common strategies such as an increase in hiding behavior or a decrease in activity lead to decreases in predator encounter rates. However, some species exhibit anti‐predator strategies that do not lead to a decrease in their encounter rate, relying more on their specific escape abilities than on morphological defenses (Lima, 1992). For open water species, avoiding structured environments can be adaptive under certain circumstances, that is, where refuges are associated with an increased predation threat or where prey escape abilities are enhanced in open water (Savino & Stein, 1989). In this study, the avoidance of the macrophyte refuge in rudd might be due to that it identified the artificial macrophytes as a possible hiding place for the predator, associate it with increased danger and therefore avoided it, although we are cautious with such an interpretation due to limited the space of the experimental tank in the chemical cue experiment. Notwithstanding, it seems that the actual change in behavior as a response to chemical cues only explains the ability of species to detect the danger, while the actual adaptive value of the behavior remains unclear until evaluated in the context of a specific predator‐prey interaction.

Each species implements a specific response to the threat of predation based on its evolutionary history. Prey species and individuals that possess morphological defenses exhibit fewer behavioral alterations in response to predation risk than species lacking such defenses (Abrahams, 1995; Andraso & Barron, 1995; Hulthén, Chapman, Nilsson, Hollander, & Brönmark, 2014). Because various morphological and behavioral adaptations form a multidimensional defense strategy, in which each species has its own anti‐predator niche (Eklöv & Persson, 1995; Whittaker, Levin, & Root, 1973), each species can possibly occupy its local optimum in the landscape of threat (Brown & Vincent, 1992). Hence, each trait is not equally important in a species‐specific anti‐predator strategy, and so comparison among species traits may not provide a reliable estimate of their anti‐predator effectiveness. Species with poor anti‐predator adaptations should try to avoid highly predated habitats, while species with well‐developed anti‐predator strategies can coexist with predators and rely on crypsis, their morphology or their escape ability (Wirsing, Cameron, & Heithaus, 2010).

In our study, only perch possesses morphological defenses, and this species did not exhibit any behavioral alterations in response to the threat of predation. However, its close relative *P. flavescens* can detect and react on chemical cue stimuli in behavioral and morphological changes (Barry, Dehnert, Hoppe, & Sorensen, 2017; Harvey & Brown, 2004; Mirza & Chivers, 2001). In contrast with *P. flavescens*, European perch was found to detect and react on chemical cues only with visual stimuli (Mikheev, Wanzenbock, & Pasternak, 2006) and has probably weaker response to chemical cues.

No species was significantly preferred by catfish in prey selectivity experiment, although perch was the most frequent prey item. However, due to limited space of the experimental setup, this experiment tests rather catfish prey preference than prey avoidance abilities based on chemical cues due to that their active range is larger than tank size (Wisenden, 2008). As no species was significantly preferred by the catfish, the observed preferences for some species in wild might be accounted for niche overlap and/or inefficient avoidance abilities of the prey rather than active prey choice (Johansson et al., 2004; Turesson & Persson, 2002; Wirsing et al., 2010). Despite their rarity, large perch were found to be a very abundant prey item in catfish larger than 80 cm, suggesting that catfish does not avoid such morphological defenses (Wysujack & Mehner, 2005). Although perch was not positively selected by catfish in another study, the predation pressure by catfish seemed to decrease its population in the long‐term study of two experimental lakes (Vejřík et al., 2017). In the same study, the most affected species by catfish predation was rudd, while roach population increased after catfish introduction to both lakes. In accordance with these results were food content and stable isotope analyses, where rudd had positive and roach negative electivity index (Vejřík et al., 2017). Catfish effectively locates moving prey in the darkness by lateral line (Pohlmann, Atema, & Breithaupt, 2004; Pohlmann, Grasso, & Breithaupt, 2001), is active in experimental lakes predominantly at night (Gjelland et al., 2017) and authors hypothesize that this is the reason why nocturnally‐active rudd becomes very frequently prey of nocturnal predators (Hölker et al., 2007; Vejřík et al., 2017).

The prey species markedly differed in their behavioral parameters in the control treatments of the chemical cue experiment. Rudd exhibited the highest shoaling and activity values, from which the latter may imply high encounter rates with predators (Lima, 1998) as well as the facilitation of the search for prey by predators when they can effectively track wakes (Pohlmann et al., 2004). However, this inter‐species comparison should be treated with caution because the fish were enclosed in a space‐limited environment, and each species may perform differently under laboratory conditions. Hence, we emphasize in this study the intra‐species differences between the experimental and control treatments, as they provide information about the ability to detect chemical cues, and avoid extrapolating the inter‐species differences to field scenarios.

The design of this study was adjusted to the logistical limitations of obtaining experimental fish from lakes Milada and Most, where catfish impact on the community as well as its selectiveness to tested prey species was evaluated (Vejřík et al., 2017). Instead, we used fish from a pond where community structure was similar and where the main predators were catfish and pike as in the lakes Most and Milada (Vejřík et al., 2017). Therefore, we assumed that experimental fish had similar reaction to chemical cues as fish inhabiting studied lakes. The setup of chemical cue experiment was simplified to twilight conditions, while fish face the whole range of light conditions depending, for example, on the day period and depth of the habitat. Future studies using, for instance, 3D telemetry tracking of predator and prey species may shed more light on actual habitat overlap and optimal avoidance strategies (Gjelland et al., 2017).

The majority of previous studies have focused on assessing the importance of chemical cues in fish survival under laboratory conditions (Ferrari et al., 2010; Mathis & Smith, 1993a). However, Pettersson et al. (2000) showed that the active range of a 50 ml of dietary stimulus prepared as above may still be detected after dilution to the equivalent of 21 m^3^. It was further shown that chemical cues are perceived below the threshold level and that fish then increase vigilance toward secondary cues during local risk assessment (Brown et al., 2004). A field experiment demonstrated that an alarm stimulus from 2 cm^2^ of skin can be subsequently detected within a range of 2–8 m from the source (Wisenden, 2008). However, despite a growing body of field evidence, a link between an ability to perceive chemical cues and the probability of prey survival in the field is still missing.

Although olfactory detection of chemical cues is not the only option for gaining an informational advantage over predators, it has recently been shown to be widespread and has been hypothesized as a crucial structuring force in aquatic ecosystems. In the experimental setup, roach exhibited most pronounced behavioral changes in response to catfish cues, and this could theoretically explain its low susceptibility to catfish predation (Vejřík et al., 2017). Because this study provides only a hint of particulars of chemical cues perception and species‐specific vulnerability to predation, we propose that more field evidence should be collected to test whether laboratory findings have repercussions in the natural environment.

## CONFLICT OF INTEREST

None Declared.

## AUTHOR CONTRIBUTIONS

JP, CB, LV, and MŠ designed the study. MŠ, ZS, MČ, PB, LV, IV, MV, and MP performed experiments. PB, DR, and ATS performed statistical analysis. MŠ wrote the first draft. All authors contributed with substantial comments during manuscript preparation.
